# Correlation between serum triglycerides and prostate cancer risk in Chinese men: a meta-analysis

**DOI:** 10.3389/fonc.2026.1784445

**Published:** 2026-04-10

**Authors:** Yuan Liu, Fei Wei, Yongxiang Yi, Ting Liu

**Affiliations:** 1 Department of Urology, The Third Affiliated Hospital of Zhejiang Chinese Medical University, Hangzhou, Zhejiang, China; 2 Department of Pathology, Beijing Ditan Hospital, Capital Medical University, Beijing, China

**Keywords:** correlation, meta-analysis, prostate cancer, risk, triglycerides

## Abstract

**Background:**

Epidemiological research presents inconsistent findings on the association between serum triglyceride levels and the risk of developing prostate cancer. However, a systematic review and meta-analysis study on the relationship between triglyceride levels and the risk of prostate cancer in Chinese men are limited.

**Methods:**

We explored PubMed, Wanfang, and the China National Knowledge Infrastructure (CNKI) databases to find related retrospective studies on the link between serum triglycerides and prostate cancer. The Newcastle-Ottawa Scale was employed to assess the quality of the study. To compute the pooled odds ratios (OR), a random effects model was utilized.

**Results:**

Twelve studies were included, comprising 4732 cases. For prostate cancer, the OR for serum triglyceride levels were 1.03 (95%CI:0.755-1.404, P=0.898). Furthermore, subgroup analysis indicated no link between serum triglyceride levels and prostate cancer risk.

**Conclusion:**

We observed no relationship between serum triglyceride and the likelihood of prostate cancer in Chinese men. Due to wide confidence intervals, these results require cautious interpretation and confirmatory replication.

## Introduction

Prostate cancer, a malignant tumor of the prostate epithelium, is the second most common cancer in men globally and the fourth most common cancer. In China, although the incidence of prostate cancer is relatively low, with social development and changes in people’s lifestyles, the incidence and mortality rates of this disease are on the rise (1). In recent years, with the development of population aging, changes in diet composition and lifestyle and work style, and the popularization of medical examination, the detection rate of prostate cancer in the world has shown an obvious upward trend (2).

Lipids are essential substances in the human body with vital physiologi-cal functions. Both excessively high and low levels of lipids in the blood may adversely affect health, potentially leading to conditions like coronary artery atherosclerosis. Elevated triglyceride levels increase the occurrence of oxidative stress and the production of reactive oxygen clusters, thus promoting prostate cancer (3). It can directly affect the pancreas, raising the levels of oxygen free radicals in the body, which results in cellular DNA damage and tumor induction (4). During cancer development, the excessive proliferation of cancer cells may trigger abnormal lipid metabolism, which could consequently impact blood lipid and lipoprotein levels in cancer patients (5). Many studies have investigated the connection between serum triglyceride levels and prostate cancer risk, but the outcomes differ significantly. Research on prostate cancer has shown that triglyceride levels are negatively correlated with the disease in some studies (6, 7). Some studies have found a positive correlation between triglycerides and prostate cancer (8, 9). Other studies have found no significant association (10–14). Numerous studies have shown that the effect of blood lipids on prostate cancer risk varies significantly across racial groups (15). Epidemiological studies exploring the relationship between triglycerides and prostate cancer are quite extensive, but the conclusions are not entirely consistent. The main trends suggest that elevated serum triglycerides are more likely associated with poor prognosis (e.g., recurrence, advanced stage, mortality) in prostate cancer than with overall disease risk, and these findings vary across different populations. Since existing literature has conducted meta-analyses on the correlation between triglycerides and prostate cancer risk through globally published articles, indicating no correlation between triglycerides and prostate cancer risk due to potential genetic differences among populations, so I wanted to investigate whether there are differences in the correlation between high triglycerides and prostate cancer risk in the Chinese men. Therefore, based on this inconsistency, we conducted a meta-analysis of 12 retrospective studies involving 4732 patients in the Chinese men to evaluate the correlation between triglycerides and prostate risk.

## Materials and methods

### Methods

The reporting methodology for this systematic review and meta-analysis adhered to the PRISMA checklist (16).

### Systematic search strategy

Our extensive review of related studies, using the PubMed, Wanfang, and China National Knowledge Infrastructure (CNKI) databases up to July 2025, examined the correlation between serum triglycerides and the risk of prostate cancer. The keywords included: ‘triglycerides, ‘ ‘serum lipid, ‘ ‘metabolic syndrome, ‘ ‘prostate cancer, ‘ and ‘China, ‘ ‘Chinese.’ Publications were restricted to English and Chinese languages. Additionally, the study examined the references cited in the publications to identify further pertinent research.

### Study selection

To meet the inclusion criteria, studies must adhere to the following standards: 1)retrospective studies; 2) the exposure factor is serum triglycerides; 3) the study subjects are prostate cancer; 4) the study area is the Chinese men; 5) provide odds ratio[OR]) and its 95% confidence interval (CI) (or data that can calculate these). Exclusion criteria: 1) studies unrelated to systematic reviews; 2) studies difficult to obtain (full text not retrievable); 3) studies with inconsistent or untraceable data; and 4) studies involving non-Chinese men.

### Data extraction and quality assessment

Retrieve the following information from each study: the first author, the year of publication, the number of prostate cancer cases, the baseline age of participants, and the relative risk or risk ratio with its 95% confidence interval (CI). Through screening the studies, it was found that all studies were in the form of OR, so we extracted OR and their confidence intervals from all reported studies. We took the natural logarithm of the OR value of each study to obtain log OR. At the same time, calculate the standard error of log OR based on its confidence interval and extract the OR value and 95% CI obtained from the regression analysis in the original study. Merge and calculate the log OR and its standard error for each study. Convert the final merged log OR to its exponential form to obtain the final combined effect size. Two reviewers systematically extracted data using standardized tables, and any disputes were resolved through discussions among the authors. If several risk estimates are available, choose the one that best controls for potential confounding factors. The Newcastle–Ottawa Quality Assessment Scale (NOS) criteria were used to evaluate the methodological quality of each study included (17). It comprised three components: 1) selection of subjects 0–4; 2) comparability of subjects 0–2; and 3) clinical outcome 0–3. NOS scores ranged from 0 to 9.

### Statistical analysis

Meta-analysis was performed using Review Manager (RevMan) software(version 5.3). For dichotomous outcomes, the pooled treatment effect was expressed as OR with a 95% CI. Heterogeneity across the included studies was assessed using the Cochran’s Q test (with a significance level of P < 0.10) and the I² statistic. The I² statistic quantifies the proportion of total variation in study estimates that is attributable to heterogeneity rather than chance, with values of 25%, 50%, and 75% typically representing low, moderate, and high heterogeneity, respectively. A significant Q value indicates heterogeneity, whereas the I² statistic quantifies the percentage variation in effect estimates due to heterogeneity rather than sampling error, with a significance threshold of 50% (18, 19). The I^2^ index quantifies the proportion of heterogeneity between groups. When no significant statistical heterogeneity (I²<50%) is observed among the studies, a fixed-effects model is used for meta-analysis. If significant heterogeneity (I²≥50%) is present, a random-effects model is utilized for the meta-analysis. The sources of heterogeneity are further analyzed through subgroup analysis. The stability of results was evaluated using sensitivity analysis; publication bias was assessed using funnel plots and Egger’s test. A P <0.05 indicated statistically significant differences (20).

## Results

### Literature retrieval process and results

**Figure 1:**
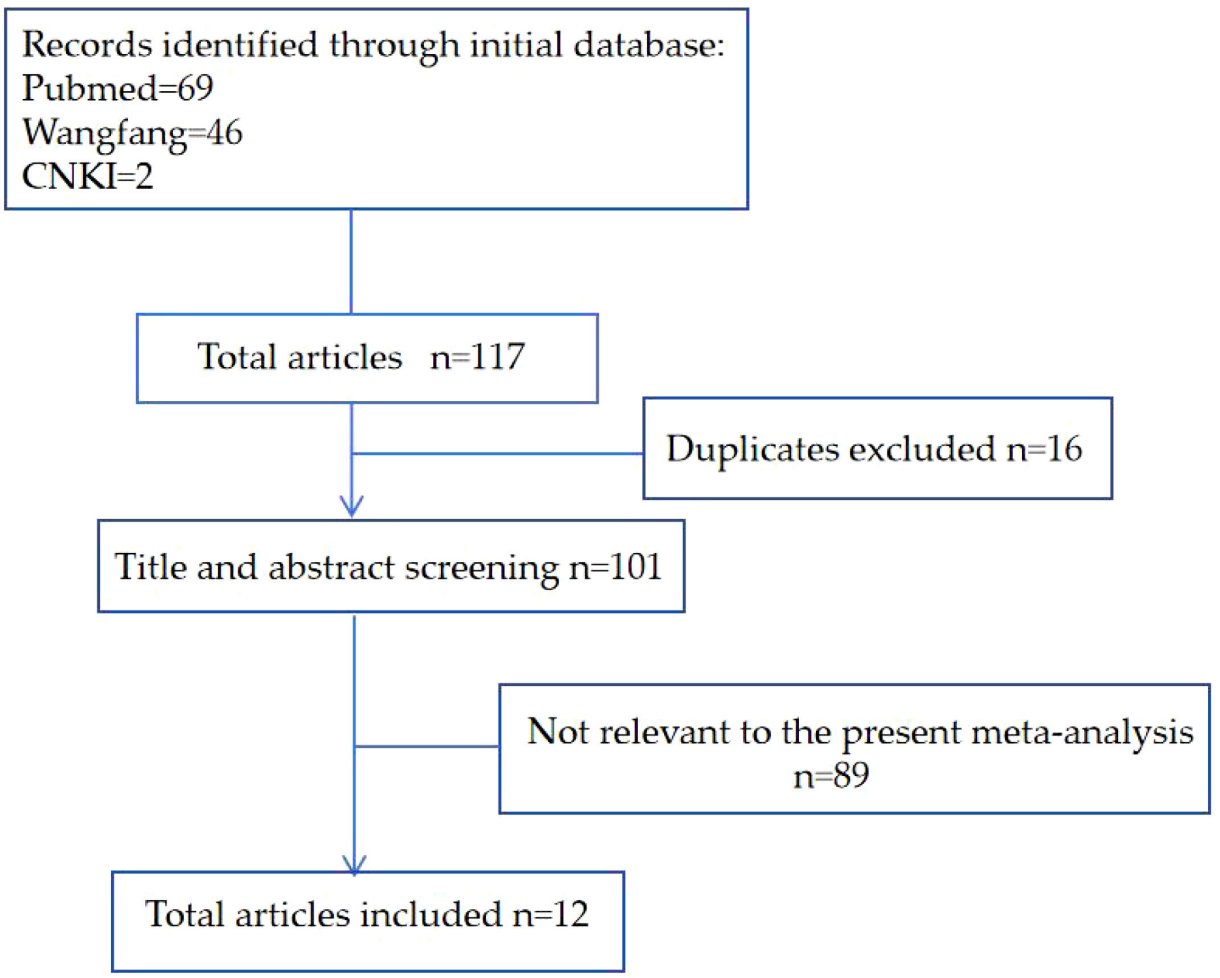
Diagram of the literature review and study selection process.

PubMed retrieved 69 articles, Wanfang retrieved 46 articles and CNKI retrieved two articles. After removing 16 redundant articles, the remaining literature was screened by reviewing titles and abstracts to exclude non-relevant works, ultimately, 12 studies involving 4732 prostate cancer patients were included, comprising six studies for English-language and six studies for Chinese language publications. The quality score of research ranges from 5 to 8 on the basis of NOS standard (**Figure 1**). This twelve articles included the first author, publication year, region, number of patients, age, and OR value (**Table 1**).

**Table 1:** Features of the studies that were included.

The first author	Year	The cases of patients	Age	China	OR	95%CI NOS	NOS
Xiaoshuai Gao (21)	2022	32	44-85	South	0.857	0.54-1.36	7
Fu Feng (22)	2023	174	NA	South	2.462	1.299-4.667	5
Jinru Wang (23)	2024	209	67.19 ± 0.62	North	2.292	1.419-3.702	6
Ke Bu (24)	2025	746	78.36 ± 9.02	North	1.45	1.26-1.66	7
Qian Gui (25)	2025	1246	71.87	South	1.344	1.201-1.503	7
Fei Zhou (26)	2024	218	75	North	1.826	1.031-3.231	6
Qiang Fu (27)	2018	47	NA	North	0.186	0.1060-0.2383	5
Fei Li (28)	2021	1259	NA	South	0.782	0.651-0.939	5
Bo Kong (29)	2014	323	50-90	North	0.738	0.578-0.941	7
Tingchun Wu (30)	2023	260	62-89	North	0.563	0.240-1.320	6
Zhensong Xiang (31)	2019	117	72.56 ± 7.91	South	0.961	0.633-1.458	6
Jianqin Zhang (32)	2014	101	73.48 ± 3.50	North	2.91	1.612-5.241	8

### Meta-analysis result and subgroup analysis

**Figure 2:**
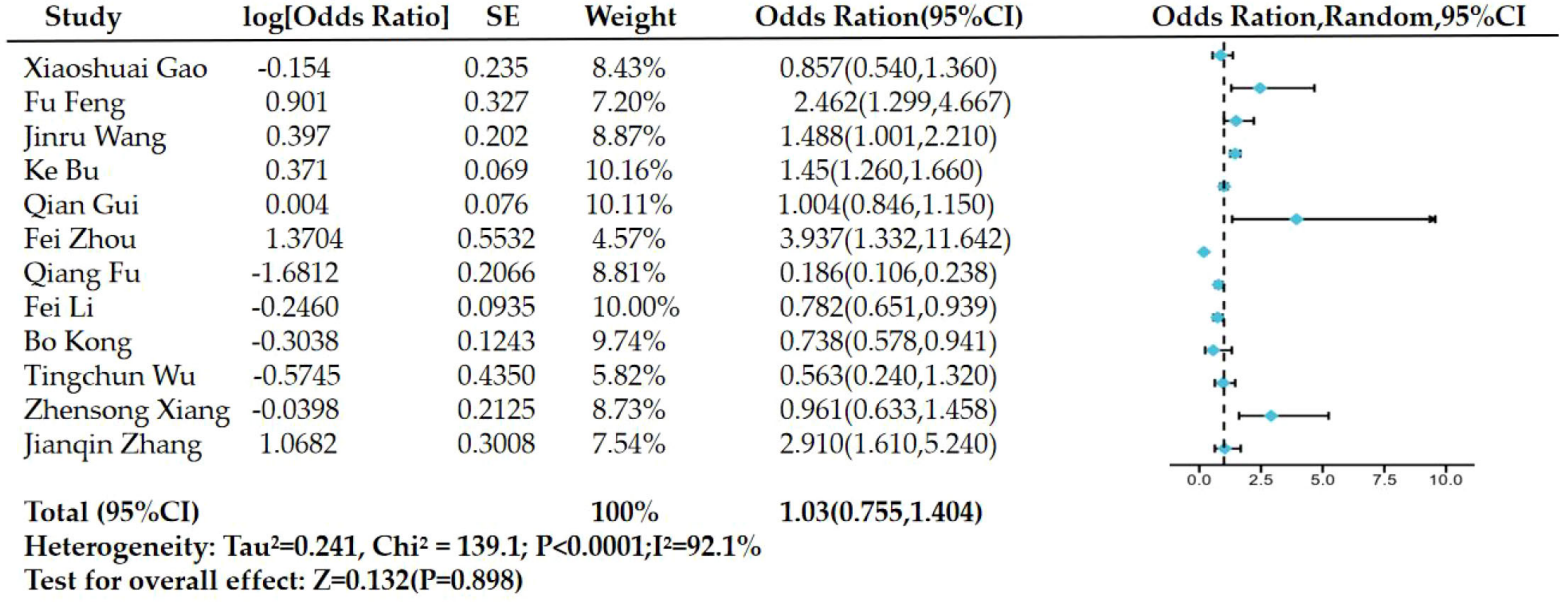
Forest plot for examining the correlation between triglycerides and prostate cancer risk.

Significant heterogeneity was observed in the 12 cohort studies included in this research (Q-test P<0.0001, I^2^=92.1%). Therefore, for the meta-analysis, a random effects model was utilized, yielding a combined effect size (OR:1.03, 95%CI:0.755-1.404), but not statistically significant(Z = 0.132, P = 0.898). The meta-analysis showed no correlation between serum triglyceride levels and prostate cancer risk in the Chinese men (**Figure 2**). We performed sensitivity analysis using the leave-one-out method. The result showed that after removing each study one by one, the I² remained between 85.4% and 92.8%, indicating that the high heterogeneity was not driven by a single study, but rather by systematic differences among studies. This indicated that regardless of whether a study was removed, the final conclusion of the meta-analysis remained unchanged. From this perspective, the conclusion regarding ‘no statistically significant difference’ is relatively stable. After excluding any single study, the pooled OR ranged from 0.96 to 1.2, with all 95% confidence intervals including 1 (no statistical significance), consistent with the original pooled result (OR = 1.030). This indicated that no single study had a decisive impact on the overall conclusion, and the results are relatively robust.

Due to limitations in the original data, we hypothesized that heterogeneity might stem from regional differences, publication year, age, PSA, and BMI. The results indicated that regional differences were the primary source of heterogeneity (p = 0.03), accounting for approximately 42% of the heterogeneity. The effect size (logOR) in northern studies was generally higher than that in southern studies. Although PSA did not reach statistical significance (p = 0.15), it explained 31.7% of the heterogeneity, likely due to insufficient statistical power caused by a larger number of missing data. Other covariates (year, age, BMI) had limited explanatory power for the heterogeneity (**Table 2**).

**Table 2:** Results of meta-regression analysis.

Cancer	Heterogeneity sources	Coef	P value	R²(%)	95%CI	
					Lower	Upper
Prostate cancer	Publication year	-0.05	0.32	8.5	-0.15	0.05
	Region(North vs South)	-0.72	0.03	42.3	-1.35	-0.09
	Age	0.02	0.68	2.1	-0.08	0.12
	BMI	-0.1	0.52	0	-0.45	0.25
	PSA	0.006	0.15	31.7	-0.002	0.014

### Publication bias

**Figure 3:**
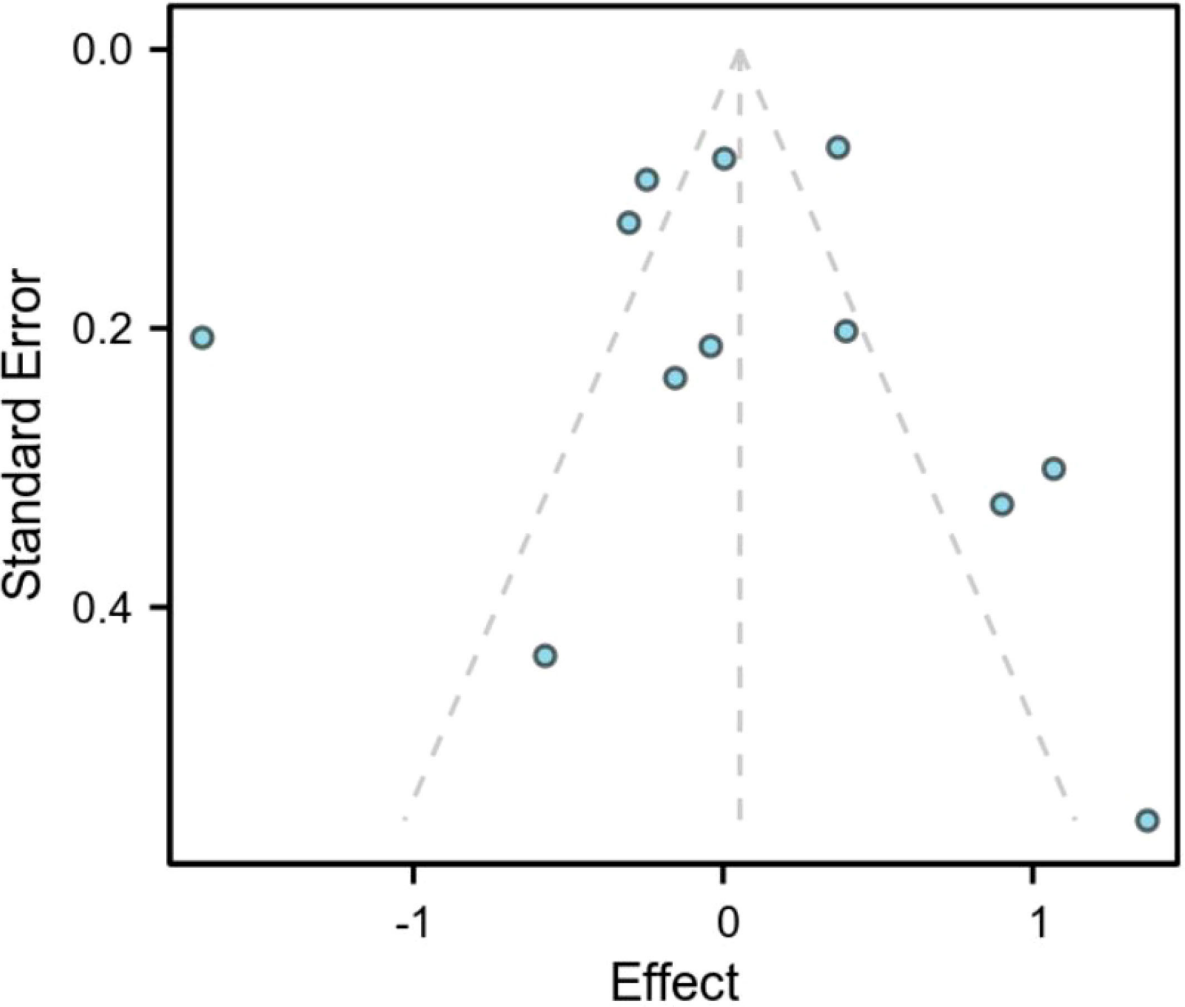
Begg’s funnel plot for investigating the link between serum triglycerides and prostate cancer risk.

We conducted Begg’s funnel plot and Egger’s test to assess publication bias in the included studies. The funnel plot revealed slight asymmetry, but the Egger’s test revealed that publication bias was not significantly present (all P-values>0.05) (**Figure 3**). The Egger test showed no statistical significance, indicating that there was no significant publication bias.

## Discussion

Prostate cancer is a prevalent malignancy in the urogenital system among men worldwide. According to data from the International Agency for Research on Cancer (IARC), there were approximately 1.414 million new cases and 375, 000 deaths from prostate cancer globally in 2020. In older men, this cancer has become the second most common form of malignancy and is the fifth leading cause of death (33). The occurrence and evolution of prostate cancer is a multi-factor and complex process, and there is no unified conclusion about this process in the world (34–37). Age, race, blood lipids, and androgens are currently considered to be linked to the occurrence and development of prostate cancer, and all of them are factors affecting prostate cancer. The prospective experimental study by O′neill demonstrated a positive correlation between hypertriglyceridemia and the development and progression of prostate cancer. This mechanism may involve abnormal serum triglyceride levels regulating oxidative stress processes, ultimately driving the transformation of normal tissues into tumor tissues (38). Foreign literature reports that consuming foods high in animal fat and oils alters lipid metabolism, triggering the production of more oxidative stress substances. This increases genetic mutations and overexpresses prostate cancer genes, significantly raising the risk of prostate cancer and mortality (39–41). However, Ma et al. conducted a meta-analysis of risk factors for prostate cancer, synthesizing data from 13 prospective studies. Their findings revealed no correlation between elevated triglyceride levels and hypercholesterolemia with the development or progression of prostate malignancies (42). The correlation between triglycerides and risk factors for prostate cancer in the Chinese men has also been less studied.

In our study, we integrated data from 12 prospective studies of the Chinese men. The results showed that there was no correlation between triglycerides and prostate cancer in the Chinese men. Studies have shown that high triglycerides can lead to changes in the hormone environment induced by fat, which in turn induces oxidative reactions or high levels of insulin-like growth factor. Fat intake can promote the proliferation of prostate cancer cells *in vivo* and *in vitro*, thereby increasing the incidence of prostate cancer. *In vitro* experiments demonstrate that triglyceride-rich residual particles induce carcinogenesis by activating key cellular signaling pathways, including MEK/ERK and Akt. These pathways regulate critical cellular processes such as growth and proliferation, apoptosis, cell cycle arrest, and lipid biosynthesis (43, 44). However, epidemiological studies have yielded conflicting findings regarding the relationship between serum triglycerides and prostate cancer risk. Some researchers suggest that studies indicate a negative correlation between triglyceride levels and prostate cancer risk (45). Some scholars believe that triglycerides and prostate cancer are positively correlated, while others report no correlation between the two (46, 47). Some scholars collected 12 prospective studies from other countries through meta-analysis and revealed no correlation between triglycerides and the risk of prostate cancer. We searched for data from 12 prospective studies in China and also confirmed this conclusion.

Evaluating the presence, extent, and sources of heterogeneity among studies is a crucial and distinctive objective of meta-analysis. Given the high heterogeneity in prostate cancer research, we conducted a meta-regression analysis for Chinese men. The results indicated no significant heterogeneity among the studies. The meta-analysis showed no statistically significant association between triglycerides and the risk of prostate cancer, and the confidence intervals were wide, suggesting limited estimation accuracy. This imprecision may stem from the following reasons: first, some included studies had small sample sizes, leading to larger standard errors; second, there was high heterogeneity among studies, and while the random-effects model is more reasonable, it also widened the confidence intervals. Sensitivity analysis indicated that the intervals narrowed slightly after removing one study, but the overall trend remained unchanged. Therefore, the current conclusions should be interpreted with caution, and more large-sample, homogeneous studies are needed in the future to provide more accurate effect estimates. Subgroup analysis was subsequently conducted to identify sources of heterogeneity, revealing that geographical region was the primary source of heterogeneity (p = 0.03), accounting for approximately 42% of the heterogeneity. The effect size (logOR) in northern studies was generally higher than that in southern studies. We also employed the Beggar’s funnel plot and Egger’s test to analyze the risk of publication bias in the studies that were included. While the Beggar’s funnel plot indicated slight asymmetry, the Egger’s test (based on specific data) showed no significant publication bias (all test P-values>0.05). Sensitivity analyses performed by sequentially excluding individual studies demonstrated that none of the individual studies significantly affected the overall meta-analysis results.

The core innovation of this article lies in its first exploration of the correlation between triglyceride levels and the risk of prostate cancer in Chinese men. By utilizing data from several studies and applying rigorous statistical approaches, this research thoroughly examined the relationship between triglycerides and prostate cancer among Chinese men. To ensure the reliability of the research findings, only high or moderate quality studies were included in the analysis. However, this study has potential limitations. Firstly, since the research strategy only included studies published in English or Chinese, selection bias was inevitable. Secondly, our focus was on published literature, and the lack of access to some might lead to publication bias. Consequently, future research with thorough data is required to validate these findings. Thirdly, this study found no correlation between triglyceride levels and the risk of prostate cancer in Chinese men, but due to the wide confidence interval, the conclusions of this study may be influenced by the characteristics of the included studies and are therefore somewhat sensitive. Further high-quality, large-sample studies are needed to confirm these conclusions. Finally, this study lies in the significant clinical and methodological heterogeneity among the original studies, as well as the inconsistency in effect measurement. This restricts our ability to precisely quantify the results and suggests that readers should exercise caution when interpreting these conclusions. However, this heterogeneity precisely reveals the current lack of unified, standardized measurement tools and reporting norms in this field. Therefore, the core value of this study lies not only in providing a preliminary estimate of the pooled effect, but also in revealing a crucial academic gap: there is an urgent need for large-sample, standardized prospective cohort studies, using consistent outcome measures.

In conclusion, although the analysis results indicated no correlation between serum triglyceride levels and prostate cancer risk, due to the wide confidence interval and significant heterogeneity, any inference regarding the intervention effect should be made with extreme caution. Future research is necessary to further verify this hypothesis through a larger sample size and a more standardized design, in order to narrow the confidence interval and explore the sources of heterogeneity.

## Data Availability

The original contributions presented in the study are included in the article. Further inquiries can be directed to the corresponding author.
